# Fluorescent Transgenic Zebrafish *Tg(nkx2.2a:mEGFP)* Provides a Highly Sensitive Monitoring Tool for Neurotoxins

**DOI:** 10.1371/journal.pone.0055474

**Published:** 2013-02-01

**Authors:** Xiaoyan Zhang, Zhiyuan Gong

**Affiliations:** Department of Biological Sciences, National University of Singapore, Singapore, Singapore; Mayo Clinic, United States of America

## Abstract

Previously a standard toxicological test termed as DarT (*Danio rerio* Teratogenic assay) using wild type zebrafish embryos has been established and it is widely applied in toxicological and chemical screenings. As an increasing number of fluorescent transgenic zebrafish lines with specific fluorescent protein expression specifically expressed in different organs and tissues, we envision that the fluorescent markers may provide more sensitive endpoints for monitoring chemical induced phenotypical changes. Here we employed *Tg(nkx2.2a:mEGFP)* transgenic zebrafish which have GFP expression in the central nervous system to investigate its potential for screening neurotoxic chemicals. Five potential neurotoxins (acetaminophen, atenolol, atrazine, ethanol and lindane) and one neuroprotectant (mefenamic acid) were tested. We found that the GFP-labeled ventral axons from trunk motoneurons, which were easily observed in live fry and measured for quantification, were a highly sensitive to all of the five neurotoxins and the length of axons was significantly reduced in fry which looked normal based on DarT endpoints at low concentrations of neurotoxins. Compared to the most sensitive endpoints of DarT, ventral axon marker could improve the detection limit of these neurotoxins by about 10 fold. In contrast, there was no improvement for detection of the mefenamic acid compared to all DarT endpoints. Thus, ventral axon lengths provide a convenient and measureable marker specifically for neurotoxins. Our study may open a new avenue to use other fluorescent transgenic zebrafish embryos/fry to develop sensitive and specific toxicological tests for different categories of chemicals.

## Introduction

The zebrafish (*Danio rerio*) has been an increasingly popular experimental model in biological research in the past two decades, not only in developmental biology but also in medical research. The zebrafish model has many advantages in laboratory research, e.g. transparent embryos, high fecundity with hundreds of embryos from each single spawning on a daily basis, low cost and space requirement for aquarium maintenance, etc. As a vertebrate model, the zebrafish offers more relevant information to human health than invertebrate models such as Drosophila and Caenorhabditis elegans [1]. Compared to *in vitro* cell based studies, the zebrafish serves as an authentic *in vivo* model in whole-organism physiological context. The value of the zebrafish model has also been increasingly recognized in toxicology and environmental science [2].

Now the zebrafish also emerges as an excellent toxicological model. In 2002, Nagal has described a standard DarT (*Danio rerio* Teratogenic assay), in which wild type zebrafish embryos are used to monitor several lethal and sublethal endpoints for evaluating the potential toxicity of chemicals at different developmental stages, and the assay covers essentially all major organs and systems in zebrafish [3]. Since then, it has been an established zebrafish embryo test recommended by OECD (Organisation for Economic Co-operation and Development) and it is also widely used in chemical screening [4]. It is very convenient to screen zebrafish embryos/larvae in a microtiter plate with a small quantity (i.e., mg/L, µg/L) of candidate chemicals. Moreover, it has the potential to develop medium- to high-throughput screening platforms with embryos/larvae in a single well of standard 6-, 12-, 24- or 96-well plates [5]. In recent years, the zebrafish has also been increasingly used as a predictive model for assessing drug-induced toxicity, including cardiotoxicity, hepatotoxicity, neurotoxicity and developmental toxicity assessment [4], [6], [7], [8], [9].

GFP or other fluorescent protein transgenic zebrafish have played an important role in developmental analyses as the fluorescence-labeled tissues and organs can be conveniently monitored in live embryos/larvae throughout the early development [10], [11]. Now there are a large number of fluorescent transgenic zebrafish lines available and these transgenic zebrafish lines, including enhancer/gene trapped lines [12], [13], have been targeted for fluorescent protein expression in essentially all tissues and organs. We envisage that the fluorescence-labeled tissues/organs may provide a more sensitive marker than wild type embryos/fry in toxicological and teratogenic tests. In order to explore the potential of fluorescent transgenic zebrafish in toxicological tests, in the present study, we selected a GFP transgenic zebrafish line, *Tg(nkx2.2a:mEGFP)*, in which GFP gene expression under the *nkx2.2a* promoter is specifically in the central nervous system (CNS) and pancreas [14], [15], [16], [17]; thus, this transgenic line may be suitable for testing chemicals with neurotoxicity. To test our hypothesis, we selected five neurotoxin chemicals of different modes of action, acetaminophen, atenolol, atrazine, ethanol and lindane (hexachlorocyclohexane), and one neuroprotectant, mefenamic acid. After exposure of these chemicals to *Tg(nkx2.2a:mEGFP)* embryos/larvae at different concentrations, we found that indeed all of the neurotoxins tested caused significant shortening of GFP-labeled axons at concentrations that would not resulted in any observable changes of the lethal and sublethal markers used in DarT. Thus, our study indicates that *Tg(nkx2.2a:mEGFP)* zebrafish provides a more sensitive tool for monitoring neurotoxin chemicals than wild type zebrafish.

## Materials and Methods

### Ethics statement

All experimental protocols were approved by Institutional Animal Care and Use Committee (IACUC) of National University of Singapore (Protocol 079/07).

### Materials

Transgenic zebrafish line *Tg(nkx2.2a:mEGFP)* was kindly provided by Dr. Joan K. Heath [14], [15], [16], [17]. Six chemicals tested in the present study were purchased from various commercial sources: acetaminophen (Sigma, A7085), atenolol (Sigma, A7655), atrazine (Chem service, PS380), ethanol (Merck, 1.00983.2500), lindane/hexachlorocyclohexane (Sigma, H4500) and mefenamic acid (Sigma, M4267).

### Exposure of chemical treatment to zebrafish embryos

Homozygous *Tg(nkx2.2a:mEGFP)* were used to cross with wild type fish in order to obtain 100% transgenic embryos for chemical exposure experiments. Embryos were collected and incubated in egg water at 28°C. Following the protocol of DarT where embryos were transferred to test solutions about 60 minutes after initiation of spawning [3], we standardized the chemical exposure time at round 3 hpf by selecting alive, well developing embryos for chemical treatment, which was carried out in 6-well plates from 3 to 120 hpf. In each well, 50 embryos were placed with 5 ml of chemical solution. Each concentration was tested in parallel in different wells with up to four independent replicates. The appropriate concentrations were determined by preliminary experiments with reference to previous publications if available. Most of the selected concentrations were below LC50. During the test, chemical solutions were changed every day.

### Phenotypical observation

During the treatment from 3 hpf to 120 hpf (before the feeding stage), several lethal or sublethal endpoints based on the DarT protocol [3], including survival rates, hatching rate, edema, tail detachment, somite formation, spontaneous movement, heart beat, pigmentation and touch response were observed and recorded as indicators for chemical toxicity.

### Imaging and data analysis

GFP fluorescence was observed under a fluorescent microscope (ZEISS Axiovert 200M) with a GFP filter and photographed with a digital camera (ZEISS AxiocCam HRC). For direct comparison in the same set of experiment, images were taken for the same exposure time at a fixed aperture. At least 5 embryos/larvae were randomly selected from each dosage group and photographed. Swimming larvae were anaesthetized with 0.1% 2-phenoxyethanol prior to photography. For length measurements of whole body, central nervous system (CNS) and axon, ImageJ software was used. After setting scale for each view under each magnification in ImageJ, body length was measured as horizontal distance from the beginning of fish head to the end of tail; CNS length was measured as horizontal distance of GFP from brain to tail; ventral axon length was measured as vertical distance from the ventral edge of the spinal cord to the ventral terminal of axon labeled by GFP for all ventral motoneuron axons from somite 5 to somite 14 for each fry. For each treated group, at least 5 fry were measured for statistical calculation.

### Statistical methods

For each chemical concentration, there were four replicates and each replicate had 50 embryos. Thus, 200 embryos per chemical per dose were used. The number of embryos for each lethal or sublethal endpoint was recorded and all values were computed base on the original embryo number (200). P-value was calculated by t-test among the four replicates in comparison to respective controls. P<0.01 was considered highly significant difference and P<0.05 significant difference from control.

## Results

### Evaluation of developmental toxicity with selected DarT endpoints

**Figure 1:**
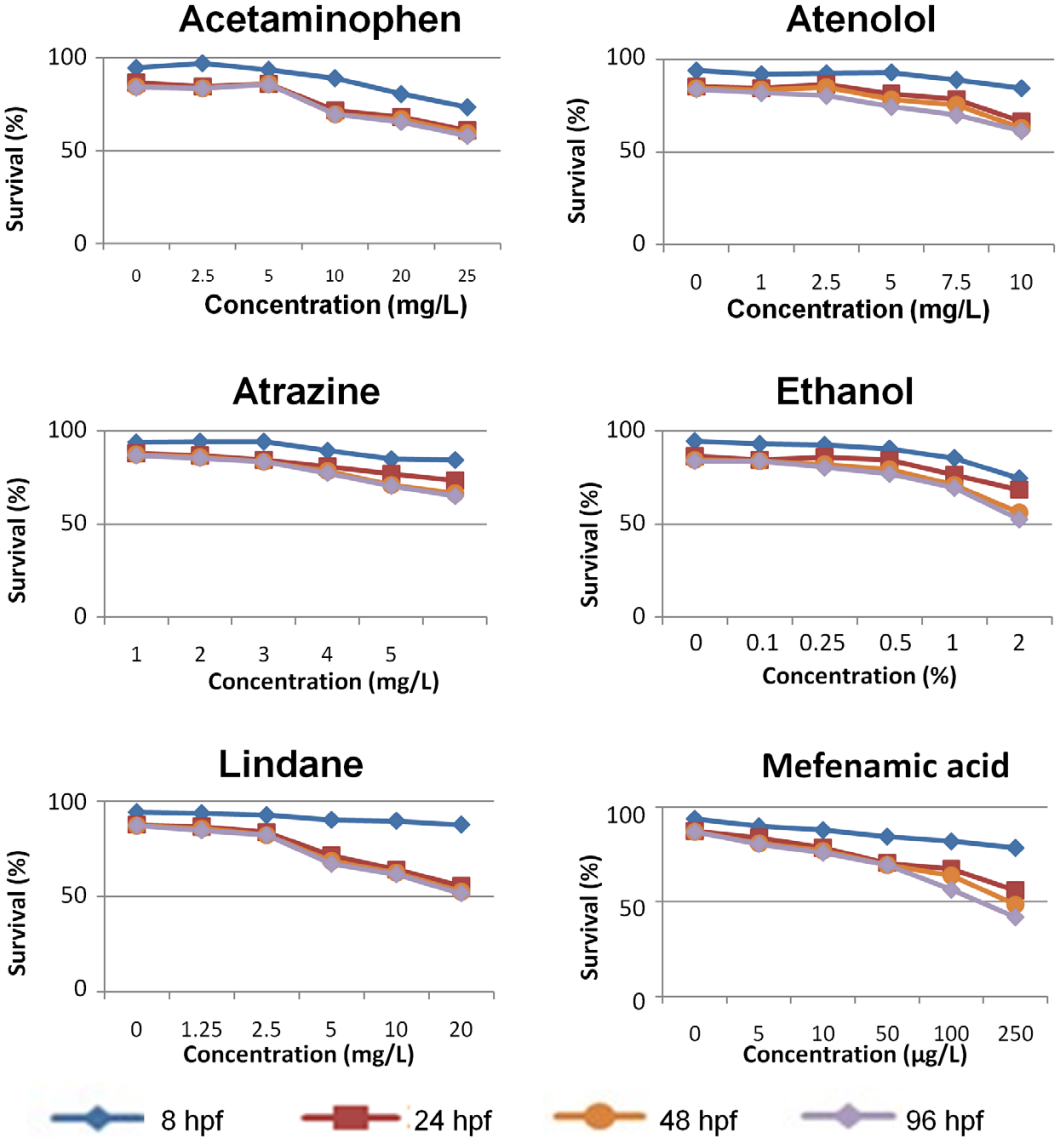
Survival rates of *Tg(nkx2.2a:mEGFP)* fry in the presence of different concentrations of testing chemicals. Survival rates at 8, 24, 48 and 96 hpf were plotted against different concentrations of the chemicals. Chemical names are indicated above each panel.

**Figure 2:**
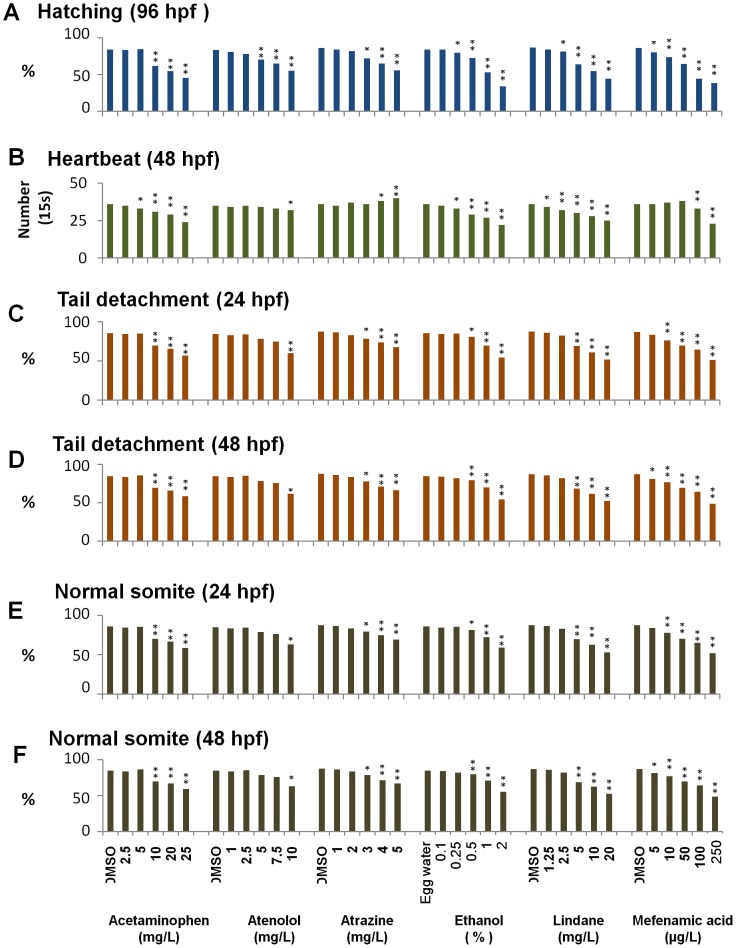
Summary of selected DarT endpoint measurements. (A) Hatching (96 hpf), (B) Heartbeat (48 hpf), (C, D) Tail detachment (24 hpf, 48 hpf), (E, F) Normal somite (24 hpf, 48 hpf). Names and concentrations of chemicals are indicated at the bottom of Panel F. 0.01% DMSO was used as control except that egg water was used as control for ethanol test. Hearbeat is shown as numbers per 15-second. Statistical significance: **P<0.01; *P<0.05.

Six chemicals with a range of five different concentrations were tested, including acetaminophen (2.5, 5, 10, 20, 25 mg/L), atenolol (1, 2.5, 5, 7.5, 10 mg/L), atrazine (1, 2, 3, 4, 5 mg/L), ethanol (0.1%, 0.25%, 0.5%, 1%, 2%,), lindane (1.25, 2.5, 5, 10, 20 mg/L), and mefenamic acid (5, 10, 50, 100, 250 µg/L) (Table S1). 0.01% DMSO was used as vehicle control for all of them except for ethanol, which is water soluble and egg water was used as the control. First, we noticed a dosage-dependent decrease of survival rates for all of the six tested chemicals at all of the four time points (8, 24, 48 and 96 hpf) (Figure 1), Generally, for all six chemicals, the survival curves for the last three time points were quite similar while the survival rates are much higher at 8 hpf, indicating that most of mortalities occurred between 8–24 hpf. For the highest dosage groups, 42.0–65.0% survival rates were observed. There was also a dosage-dependent decrease hatching rates for all six chemicals (Figure 2A), with suppression of the hatching rates to 34%–56% in their highest concentration groups. These observations further indicate the effectiveness of these chemical treatments as well as the toxicity of these chemicals.

**Figure 3:**
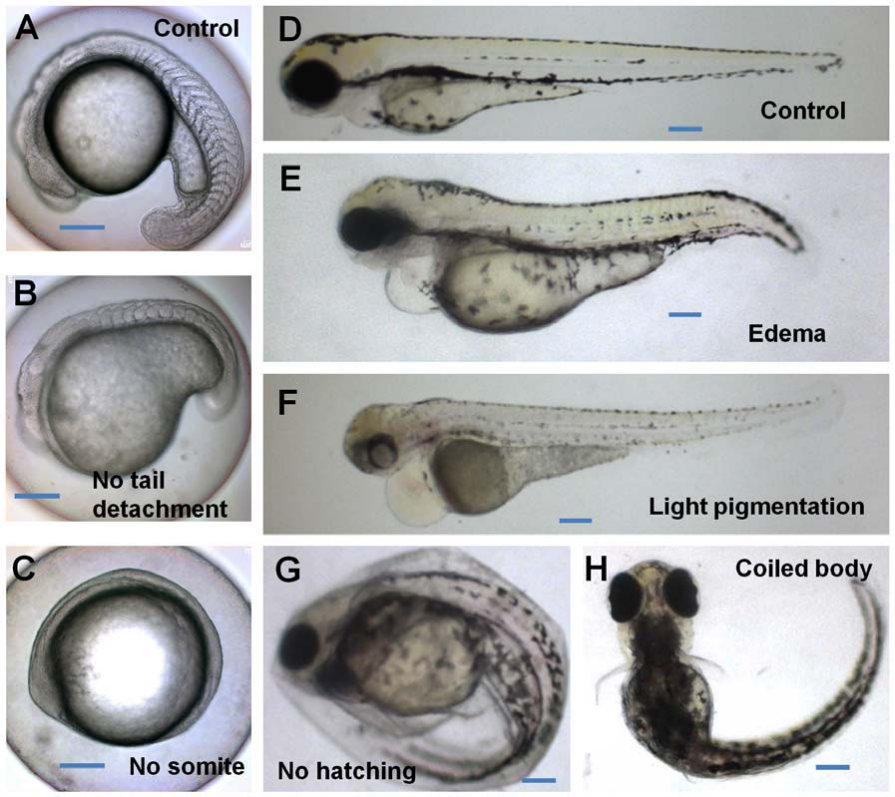
Examples of abnormal phenotypes. (A, D) Normal developing control embyors/fry in o.01% DMSO at 24 hpf (A) and 72 hpf (D); (B) No tail detachment at 24 hpf in 20 mg/L acetaminophen; (C) No somite at 24 hpf in 25 mg/L acetaminophen; (E) Edema at 72 hpf in 20 mg/L lindane; (F) Light pigmentation at 72 hpf in 250 µg/L mefenamic acid; (G) No hatching at 72 hpf in 10 mg/L lindane; (H) Coiled body at 96 hpf in 5 mg/L lindane. Scale bars: 200 µm.

We also examined several other DarT endpoints, including tail detachment and somite formation at 24 hpf and 48 hpf; spontaneous movement at 24 hpf; heart beat at 48 hpf; hatching at 96 hpf; edema, touch response and pigmentation between 90–120 hpf (Table S1). Some examples of the abnormalities are shown in Figure 3, such as no tail detachment (Figure 2B), no somite formation (Figure 3C), edema (Figure 3E), light pigmentation (Figure 3F), lack of hatching (Figure 3G), in comparison with matched controls (Figure 3A and 3D). Statistics of some of these abnormalities are presented in Figure 2 and all the DarT endpoints measured are summarized in Table S1. In general, there was a dosage-dependent effect for essentially all of the six chemicals on all these traits except for the heartbeat rates where acetaminophen, ethanol, lindane, and mefenamic acid caused dosage-dependent decrease but atenolol and atrazine treatments showed no significant change or slightly increase of heartbeat (Fig. 2B). To evaluate the significance of difference we observed, T-test was used; significant difference (P = 0.01–0.05) and highly significant difference (P<0.01) from the control groups are indicated in Figure 2 and Table S1. Highly significant differences for all or most of these endpoint measurements were observed only from high dosage groups of five of these chemicals and their starting concentrations were: 10 mg/L acetaminophen, 4 mg/L atrazine, 0.5% ethanol, 5 mg/L Lindane and 10 mg/L mefenamic acid. For atenolol, most endpoints did not show significant difference but hatching and edema appeared to be quite sensitive indicators with the highly significant difference (p<0.01) at the concentration of 5 and 7.5 mg/L respectively while most other traits did not show highly significant difference even at the highest dosage (10 mg/L) used (Table S1).

In addition, we also observed some specific effects for these tested chemicals. Atrazine had a dosage-dependent increase of heartbeat rate (but with a smaller magnitude of heart contraction) while all other five chemicals caused a dosage-dependent decrease of heartbeat (Figure 2B and Table S1). High dose ethanol led to, obvious edema with shorter body length in a high percentage of treated fry. High dose lindane generally resulted in coiled body and shorter body length; when these treated were touched, they had spiral-locally swimming pattern. For mefenamic acid, high dose groups of fry had light or no pigmentation (Figure 3F), in addition to high percentage of edema.

### Axon length provides a more sensitive and measurable marker for evaluation of neurotoxixity

**Figure 4:**
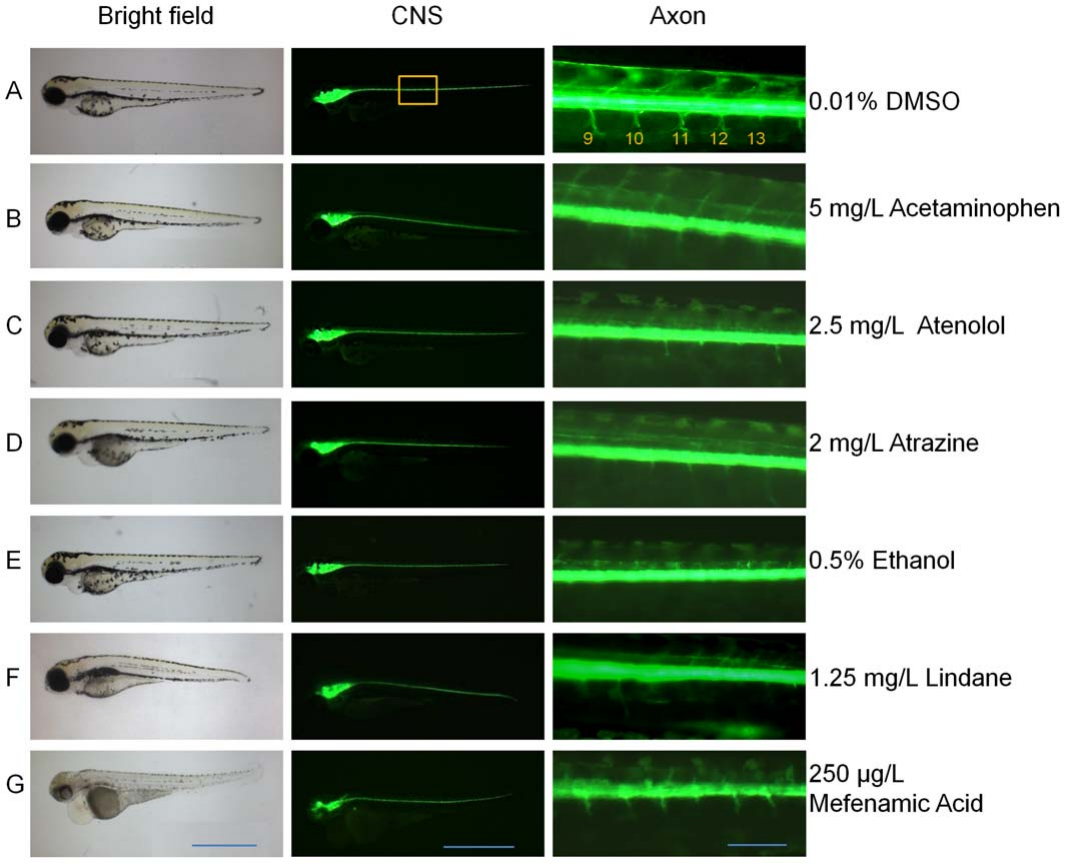
General phenotypes (left row), GFP-expressing central nervous systems (middle row) and motoneuron axons (right row) of 80-hpf *Tg(nkx2.2a:mEGFP)* fry in the presence of effective concentrations of different chemicals. (A) 0.01% DMSO control, (B) 5 mg/L Acetaminophen, (C) 2.5 mg/L Atenolol, (D) 2 mg/L Atrazine, (E) 0.5% Ethanol, (F) 1.25 mg/L Lindane and (G) 250 µg/L Mefenamic acid. Somite numbers are indicated at the top right panel. Scale bars: 1000 µm for the left and middle rows and 100 µm for the right row.

In order to demonstrate that GFP fluorescence may provide more sensitive markers for phenotypical changes induced by these chemicals, GFP fluorescence was observed and photographed for each treatment group. As reported previously [16], [17], GFP fluorescence was observed in the developing neural tube and brain from 1 dpf. By 3 dpf, obvious GFP-labeled axons were observed from motoneurons in the trunk region. As shown in Figure 4, the larvae in the control group (0.01% DMSO or egg water) had well grown ventral axons. In comparison, the ventral axons were either shortened or abolished by treatment with all of the five neurotoxins: acetaminophen, atenolol, atrazine, ethanol and lindane (Figure 4B–F). In contrast, the axons were largely unaffected by the neural protectant, mefenamic acid (Figure 4G), indicating the specific response of axon growth to neurotoxins.

**Figure 5:**
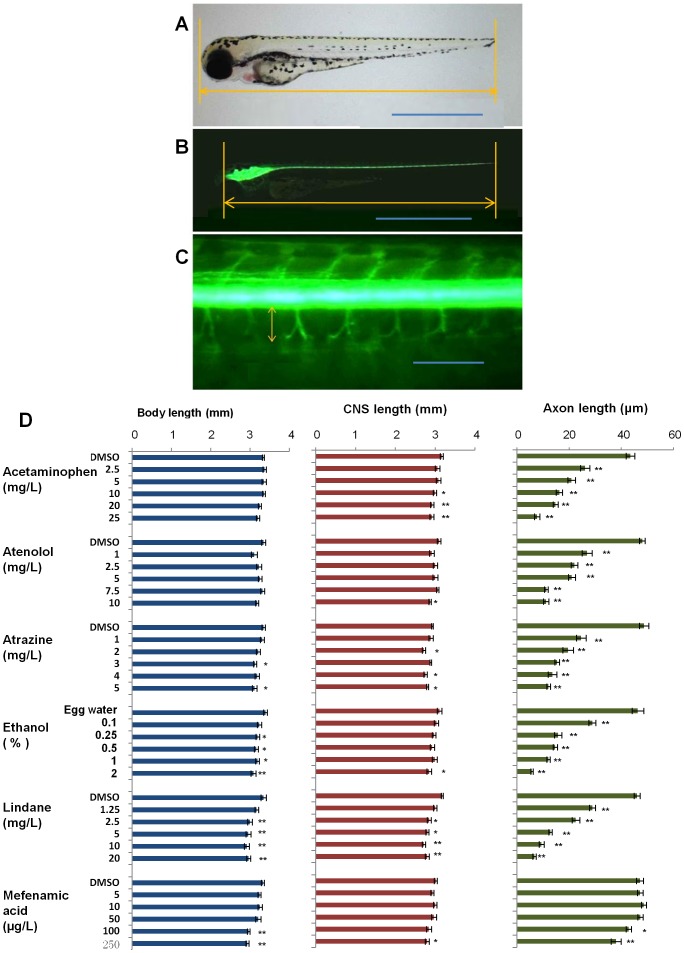
Body length, CNS length and axon length of *Tg(nkx2.2a:mEGFP)* fry in the presence of variable chemicals. (A–C) Examples of measurements of body length (A), CNS length (B) and axon length (C). The measured lengths are indicated by double arrow lines. Scale bars: 1000 µm in (A.B) and 100 µm in (C). (D) Histograms of body length, CNS length and axon length. Chemical names and concentrations are indicated on the left. Statistical significance: **P<0.01; *P<0.05.

To further evaluate the toxic effects of these chemicals, lengths of anteiro-posterior body, the central nervous system (CNS) and ventral axons were measured. Among the three lengths, only body length measurement is in wild type larvae. As shown in Figure 5 and Table S1, only high doses of atrazine, ethanol, lindance and mefenamic acid showed measureable difference (P = 0.01–0.05) compared to the control groups, but only highest concentration groups of ethanol (2%) and of mefenamic acid (100, 250 µg/L) showed statistically highly significant difference (P<0.01). For CNS length, only the two highest doses (20 and 25 mg/L) of acetaminophen showed highly significant difference (P<0.01) although other four neurotoxins, but not mefenamic acid, also resulted in measurable shortening (P = 0.01–0.05) in their high concentration groups.

In contrast, by measurement of axon length, we found that even the lowest dose of all of five neurotoxins (2.5 mg/L acetaminophen, 1 mg/L atenolol, 1 mg/L atrazine, 0.1% ethanol, 1.25 mg/L lindane) caused highly significant (P<0.01) shortening (Figure 5 and Table S1). Compared to the starting concentrations of highly significant changes observed based on standard DarT endpoints examined under a bright-field microscope, the axon length endpoint would increase detection sensitivity by at least 2–5 fold for the five neurotoxins. It is interesting to note that there is no observed axon shortening from mefenamic acid treatment except for the highest concentration groups (100 and 250 µg/L) while other general toxicological changes (e.g. survival rates, hatching, tail detachment, somite formation, edema etc) were observed at much lower concentration (10 µg/L), suggesting that the shortened axons by mefenamic acid may be a secondary effect resulted from other primary toxicities. These observations suggest that the axon length is a quite sensitive and specific endpoint for testing neurotoxicity. The axon length was generally correlated with the lack of or abnormal touch response (Table S1), which was dosage-dependent but an apparently less sensitive trait than axonal length.

**Figure 6:**
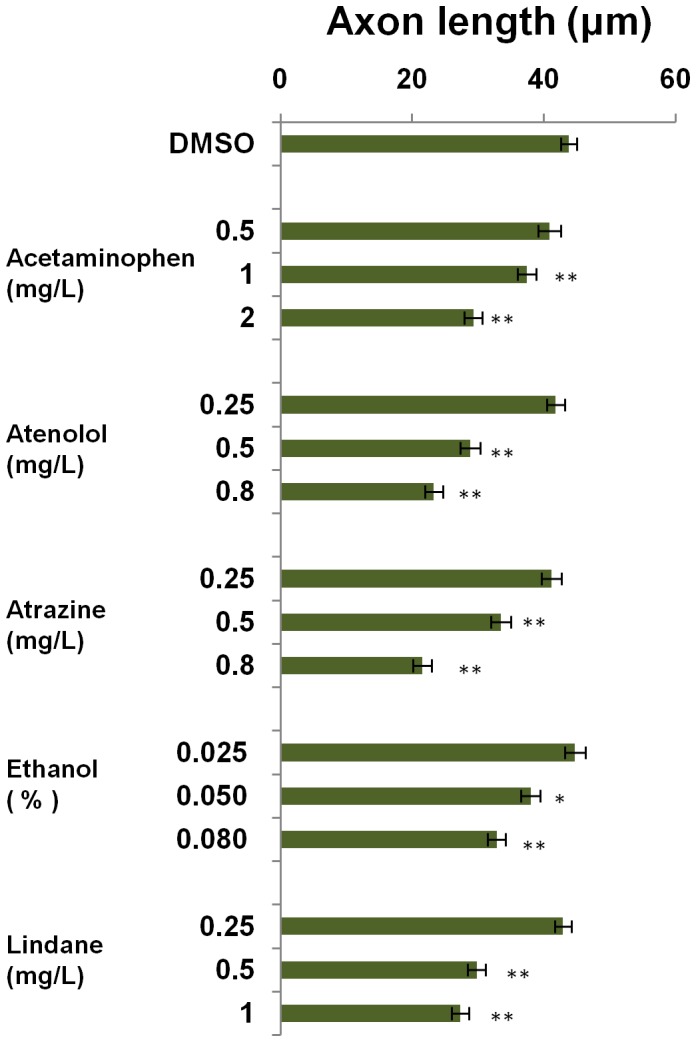
Lowest effective concentrations of neurotoxins for shortening of motoneuron axons.

To further determine the maximum sensitivity of using the axon length as a biomarker for these neurotoxins, another test with lower ranges of neurotoxin concentrations was conducted. As shown in Figure 6, highly significant difference of measured axon length (P<0.01) could be detected at the following lowest concentrations: 1 mg/L acetaminophen, 0.5 mg/L atenolol, 0.5 mg/L atrazine, 0.08% ethanol and 0.5 mg/L lindane. Thus, compared to the starting concentrations of the changes observed based on standard DarT endpoints examined under a bright microscope (10 mg/L acetaminophen, 5 mg/L atenolol, 4 mg/L atrazine, 0.5% ethanol and 5 mg/L lindane (Table S1), the axon length endpoint would increase detection of sensitivity by about 10 fold.

## Discussion

In the present study, we demonstrated that the fluorescent transgenic zebrafish *Tg(nkx2.2a:mEGFP)*, in which the central nervous system including trunk axons is marked by GFP expression under the *nkx2*.2a promoter [16], [17], can be used as a highly sensitive system for testing neurotoxins, thus providing a rapid and convenient assay to screen for neurotoxins. Previously, Fan *et al* also proposed to use a quantitative RT-PCR based assay to analyse expression of a few selected neural developmental marker genes in early zebrafish embryos for screening of neurotoxins and *nkx2.2a* is one of the markers selected [18]. Compared to the quantitative RT-PCR assay, the current fluorescent transgenic assay has directly observable and measureable phenotypes in live fry and thus is convenient and rapid. Furthermore, the fluorescent transgenic zebrafish has the potential to develop to a high-throughput assay with possible automation [19], [20]. Recently, Kanungo and colleagues have employed another transgenic zebrafish line, *Tg(hb9:GFP)*, which also expresses GFP in trunk motoneurons and axons, to develop a neurotoxin assay by measuring axon lengths and similar results have been reported for two neurotoxins, ketamine and alcohol [21], [22]. In *Tg(nkx2.2a:mEGFP)*, GFP expression appears to faithfully recapitulate the endogenous *nkx2.2a* expression in a subset of oligodendrocyte lineage [16], [17]. In both *Tg(hb9:GFP)* and *Tg(nkx2.2a:mEGFP)* transgenic lines, trunk ventral axons were used as a marker for neurotoxicity, but the GFP signal is originated from the axon per se in *Tg(hb9:GFP)* embryos while it is from the ensheathing Schwann cells in *Tg(nkx2.2a:mEGFP)* embryos. It seems that the assay with *Tg(nkx2.2a:mEGFP)* is more sensitive than that with *Tg(hb9:GFP)* as only about 20% shortening of ventral axons was reported in *Tg(hb9:GFP)* by 2% alcohol [22] while in our study with *Tg(nkx2.2a:mEGFP)* the same concentration of alcohol caused 87.7% of ventral axon reduction. Previously, it has also been reported by measuring ventral axon length for neurotoxicity evaluation by using another GFP transgenic line, *Tg(islet1:gfp)* [23], or by antibody staining of axon [24]. Thus, measurement of axon length in zebrafish embryos is being increasingly recognized as a standard assay for neurotoxins.

In this study, six chemicals with a range of dosages were tested in zebrafish embryos/larvae, including acetaminophen, atenolol, atrazine, ethanol, lindane and mefenamic acid. While two of them, ethanol [25] and lindane [26], are widely considered to be neurotoxins at high dose, three are candidate neurotoxins: acetaminophen [27], [28], atenolol [29] and atrazine [30], [31], [32]. The last one, mefenamic acid, is considered to be neuroprotectant [33]. The five neurotoxins have different molecular modes of action. Acetaminophen is a popular and over-the-counter drug for treatment of headache and its main mechanism appears to be the inhibition of cycloxygenase (COX) [34]. Atenolol is a β1-adrenoceptor antagonist while atrazine, a widely used herbicide, disrupts the photosystem II in plants by binding to the plastoquinone-binding protein [35]. Ethanol is a well known neurotoxin at high dosage through binding to acetylcholine, GABA (gamma-aminobutyric acid), serotonin, and NMDA (N-Methyl-D-aspartate) receptors [36], [37], [38]. Lindane is an organochlorine chemical used as an agricultural insecticide and it interferes with GABA neurotransmitter by interacting with the GABA receptor-chloride channel complex [39]. Despite the different molecular modes of these neurotoxins, they all inhibited axon growth in zebrafish but their inhibitory mechanisms remain unclear and will require further studies in the future. It will also be interesting to carry out chemical withdraw experiments to examine the reversibility of axon growth for further understanding of the mechanisms of these neurotoxins.

For the five neurotoxins, many studies have been conducted in experimental animals and their toxicity in the nervous system has been documented. Acetaminophen has also been previously tested in zebrafish and its general effect on embryonic development, nephrotoxicity and hepatotoxicity have been reported [27], [40], [41] but its neurotoxicity has not been studied. Its direct neurotoxic action has been recently established by both in vitro and in vivo studies in rats and neuronal apoptosis has been observed at concentration of 1–2 mM (150–300 mg/L) [28] Apparently the zebrafish larvae are more sensitive to acetaminophen as significant embryonic developmental defects were observed at concentration of 10 mg/L while significant shortening of axon length occurred at concentration as low as 2 mg/L. Atenolol may cause an allosteric inhibition of voltage-gated sodium channels and blockade of neural nitric oxide release, as reported from a study in rabbit [29]. Another study in mice shows that atenolol disrupt the positive feedback to the central nervous system and results in a decreased locomotor activity and background contextual fear [42]. Atrazine has been tested in zebrafish for developmental neurotoxicity and it increases cell death in brain and causes disorganized motor neuron axon growth [30]. Consistent with this, a mouse study has also indicated that early exposure to low doses of atrazine affects the mice behavior related to neurodevelopmental disorder [32]. Alcohol abuse and its neurotoxic effect in human have been and alcohol also causes progressive neuroinflammation and neurological disorder [43]. In zebrafish, it has been reported that ethanol causes abnormal development of motor neurons and muscle fibers [25]. The neurotoxic effect of lindane has also been well documented [26], [44] and chronic exposure of low dose lindane causes neurobehavioral, neurochemical, and electrophysiologrcal efects in rat brain [45].

Our observations in the present study are consistent with the general mode of the action of these six chemicals. All of the five neurotoxins, acetaminophen, atenolol, atrazine, ethanol and lindane, showed sensitive inhibition of axon growth. In contrast, mefenamic acid has a significant neuroprotective effect by inhibition of glutamate-induced cell toxicity *in vitro* and reduces ischemic stroke *in vivo* in rats [33]. Our observation is also consistent with its neural protectant role as the toxic concentrations (10 and 50 µg/L) of mefenamic acid, which caused statistically very significant edema, light pigmentation and shorter body length, apparently had no effect on the axon growth.

It is apparent that all of these six chemicals show dosage-dependent toxicity in essentially all the endpoints observed (Table S1). In the present study, we demonstrated that, compared to the recommended DarT endpoints, axon length, which can be observed and measured in *Tg(nkx2.2a:mEGFP)* fry, is about 10 fold more sensitive than the most sensitive endpoints recommended in DarT. Thus, with the ease and direct observable features of GFP expression, the *Tg(nkx2.2a:mEGFP)* transgenic zebrafish provides a convenient and highly sensitive tool for screening and testing neurotoxic compounds, which will be applicable in environmental monitoring and pharmaceutical production. As there are a large number of fluorescent transgenic zebrafish with fluorescent protein reporter gene expression in specific organs and tissues [10], [11], our study may open a new avenue to test other useful fluorescent transgenic zebrafish for development of specific toxicological assays for different categories of chemicals. In particular, as exampled here, all of the toxicological assays in fluorescent transgenic zebrafish can be accomplished within 5 days after fertilization and before feeding stage, which is considered an in vivo test system alternative to adult animals, thus reducing the use of animals in toxicological tests.

## Supporting Information

Table S1 **Comparison of sensitivity of lethal and sublethal DarT endpoints and axon length measurements in** ***Tg(nkx2.2a:mEGFP)*** **the treatment.**
(DOCX)
