# Supplementary material for: Fluorescent Transgenic Zebrafish Tg(nkx2.2a:mEGFP) Provides a Highly Sensitive Monitoring Tool for Neurotoxins
Source: PLoS One. 2013 Feb 1;8(2):e55474. doi: 10.1371/journal.pone.0055474 (PMC3562320; doi:10.1371/journal.pone.0055474)
Supplement: Table S1 — Comparison of sensitivity of lethal and sublethal DarT endpoints and axon length measurements in Tg(nkx2.2a:mEGFP) the treatment. (DOCX) [file pone.0055474.s001.docx]

Table S1. Comparison of sensitivity of lethal and sublethal DarT endpoints and axon length measurements in *Tg(nkx2.2a:mEGFP)* the treatment

| Time  Groups with highly significant difference (P<0.01) from controls are indicated in red and with significant difference (P<0.05) in yellow based on t-test.  Touch response: + (normal), +/- (slow response, jump), - (no response, not moving)  Pigment: + (normal), +/- (light pigmentation), - (no or little pigmentation) |  | 8 hpf | 24 hpf | | | | 48 hpf | | | | 96 hpf – 120 hpf | |  | | | *Tg(nkx2.2a:mEGFP)* | | |
| --- | --- | --- | --- | --- | --- | --- | --- | --- | --- | --- | --- | --- | --- | --- | --- | --- | --- | --- |
| Endpoint |  | Survival rate | Survival rate | Tail detached rate | With somites | With spontaneous movement | Survival rate | Tail detached rate | With somites | Heart beat (15s) | Survival rate | Hatching | Without oedema | Touch response | pigment | body length (μm) | CNS length (μm) | axon length (μm) |
| DMSO | 0.01% | 94.5 | 86.5 | 85.5 | 86.0 | 85.5 | 84.5 | 84.5 | 84.5 | 36 | 84.0 | 84.0 | 83.0 | + | + | 3331 | 3163 | 43.5 |
| Acetaminophen (mg/L) | 2.5 | 97.0 | 84.5 | 84.5 | 84.5 | 84.5 | 83.5 | 83.5 | 83.5 | 35 | 83.5 | 83.5 | 82.5 | + | + | 3364 | 3053 | 26.1 |
|  | 5 | 93.5 | 86.0 | 85.0 | 85.5 | 84.5 | 86.0 | 85.5 | 86.0 | 33 | 85.5 | 84.5 | 85.0 | + | + | 3352 | 3078 | 20.9 |
|  | 10 | 89.0 | 71.5 | 69.5 | 70.0 | 69.5 | 69.5 | 69.5 | 69.5 | 31 | 69.5 | 62.0 | 68.0 | +/- | +/- | 3350 | 2988 | 16.2 |
|  | 20 | 80.5 | 68.0 | 65.5 | 66.5 | 64.5 | 67.0 | 66.0 | 67.0 | 29 | 65.5 | 54.5 | 59.0 | +/- | +/- | 3244 | 2922 | 14.7 |
|  | 25 | 73.5 | 61.0 | 57.0 | 58.5 | 56.0 | 59.5 | 58.5 | 59.0 | 24 | 58.0 | 45.5 | 49.0 | +/- | +/- | 3197 | 2911 | 7.7 |
| DMSO | 0.01 | 94.0 | 85.5 | 84.5 | 85.0 | 84.5 | 84.5 | 84.5 | 84.5 | 35 | 84.0 | 83.5 | 83.5 | + | + | 3339 | 3093 | 48.0 |
| Atenolol (mg/L) | 1 | 92.0 | 84.5 | 83.0 | 83.5 | 83.0 | 83.5 | 83.5 | 83.5 | 34 | 82.0 | 81.0 | 81.0 | + | + | 3105 | 2914 | 26.8 |
|  | 2.5 | 92.5 | 86.5 | 84.0 | 84.5 | 83.5 | 85.0 | 85.0 | 85.0 | 35 | 80.5 | 78.0 | 78.5 | + | + | 3225 | 3000 | 22.1 |
|  | 5 | 93.0 | 81.5 | 78.5 | 79.0 | 77.5 | 78.5 | 78.5 | 78.5 | 34 | 74.5 | 70.5 | 71.5 | + | + | 3251 | 2999 | 21.0 |
|  | 7.5 | 89.0 | 78.5 | 75.0 | 76.5 | 73.5 | 75.5 | 75.5 | 75.5 | 33 | 70.0 | 65.0 | 59.0 | +/- | + | 3316 | 3061 | 11.2 |
|  | 10 | 84.5 | 66.5 | 60.0 | 63.0 | 58.5 | 63.0 | 61.5 | 63.0 | 32 | 61.5 | 55.5 | 49.0 | +/- | +/- | 3176 | 2869 | 11.2 |
| DMSO | 0.01 | 94.0 | 88.0 | 87.5 | 87.5 | 87.5 | 87.5 | 87.5 | 87.5 | 36 | 87.0 | 86.5 | 87.0 | + | + | 3341 | 2926 | 48.7 |
| Atrazine (mg/L) | 1 | 94.5 | 87.0 | 86.5 | 86.5 | 86.5 | 86.0 | 86.0 | 86.0 | 35 | 85.5 | 84.0 | 84.5 | + | + | 3305 | 2891 | 24.6 |
|  | 2 | 94.5 | 84.5 | 83.0 | 83.5 | 83.5 | 83.5 | 83.5 | 83.5 | 37 | 83.5 | 82.0 | 81.5 | +/- | + | 3204 | 2715 | 19.6 |
|  | 3 | 89.5 | 81.0 | 78.5 | 79.5 | 78.5 | 78.5 | 78.0 | 78.5 | 36 | 77.5 | 72.0 | 74.5 | +/- | +/- | 3122 | 2883 | 15.4 |
|  | 4 | 85.0 | 77.0 | 74.0 | 75.0 | 72.5 | 71.0 | 71.0 | 71.0 | 38 | 70.5 | 65.0 | 66.5 | - | +/- | 3177 | 2753 | 13.6 |
|  | 5 | 84.5 | 73.5 | 67.5 | 69.0 | 66.0 | 66.5 | 66.5 | 66.5 | 40 | 65.0 | 56.0 | 59.5 | - | +/- | 3109 | 2805 | 12.1 |
| Egg water |  | 94.5 | 86.5 | 85.5 | 86.0 | 85.5 | 84.5 | 84.5 | 84.5 | 36 | 84.0 | 84.0 | 83.0 | + | + | 3388 | 3106 | 46.3 |
| Ethanol (by volume) | 0.10 | 93.0 | 84.5 | 84.5 | 84.5 | 84.5 | 84.0 | 84.0 | 84.0 | 35 | 84.0 | 84.0 | 83.0 | + | + | 3231 | 3028 | 28.8 |
|  | 0.25 | 92.5 | 86.0 | 85.0 | 85.5 | 84.0 | 82.0 | 82.0 | 82.0 | 33 | 80.5 | 80.0 | 78.5 | + | + | 3191 | 2971 | 15.8 |
|  | 0.50 | 90.5 | 84.5 | 81.0 | 81.5 | 76.5 | 79.5 | 79.5 | 79.5 | 29 | 77.0 | 72.5 | 72.0 | +/- | + | 3154 | 2923 | 14.6 |
|  | 1 | 85.5 | 76.5 | 69.5 | 72.5 | 67.5 | 71.0 | 70.0 | 70.5 | 27 | 69.5 | 53.0 | 60.0 | +/- | +/- | 3185 | 2986 | 12.0 |
|  | 2 | 74.5 | 68.5 | 54.5 | 59.0 | 49.5 | 56.0 | 54.5 | 55.0 | 22 | 52.5 | 34.0 | 41.5 | - | +/- | 3083 | 2847 | 5.7 |
| DMSO | 0.01 | 94.0 | 87.5 | 87.5 | 87.5 | 87.5 | 87.0 | 87.0 | 87.0 | 36 | 87.0 | 87.0 | 87.0 | + | + | 3342 | 3171 | 46.0 |
| Lindane (mg/L) | 1.25 | 93.5 | 86.5 | 86.0 | 86.5 | 85.5 | 85.5 | 85.5 | 85.5 | 34 | 84.5 | 84.0 | 83.5 | + | + | 3164 | 2998 | 28.9 |
|  | 2.5 | 92.5 | 83.5 | 82.5 | 83.0 | 82.5 | 82.0 | 82.0 | 82.0 | 32 | 82.0 | 81.5 | 80.5 | +/- | +/- | 2993 | 2846 | 22.6 |
|  | 5 | 90.0 | 71.5 | 69.0 | 69.5 | 67.5 | 68.5 | 68.5 | 68.5 | 30 | 67.0 | 64.0 | 62.5 | - | +/- | 2952 | 2802 | 12.8 |
|  | 10 | 89.5 | 64.0 | 61.0 | 62.5 | 60.0 | 62.5 | 61.5 | 62.5 | 28 | 61.5 | 54.5 | 56.5 | - | +/- | 2915 | 2712 | 9.4 |
|  | 20 | 87.5 | 55.5 | 52.0 | 53.0 | 49.0 | 52.5 | 52.0 | 52.5 | 25 | 51.5 | 44.5 | 46.0 | - | +/- | 2959 | 2797 | 6.7 |
| DMSO | 0.01 | 94.0 | 87.5 | 87.0 | 87.5 | 87.0 | 87.0 | 87.0 | 87.0 | 36 | 87.0 | 86.5 | 86.5 | + | + | 3319 | 3011 | 47.1 |
| Mefenamic Acid (μg/L) | 5 | 90.0 | 84.0 | 83.5 | 84.0 | 83.0 | 81.0 | 81.0 | 81.0 | 36 | 80.5 | 80.5 | 79.5 | + | + | 3235 | 2926 | 47.2 |
|  | 10 | 88.0 | 78.5 | 76.5 | 78.0 | 76.0 | 77.0 | 76.5 | 77.0 | 37 | 76.0 | 74.0 | 75.0 | + | + | 3247 | 2999 | 48.6 |
|  | 50 | 84.5 | 70.5 | 69.5 | 70.0 | 67.0 | 69.5 | 69.5 | 69.5 | 38 | 69.5 | 64.5 | 66.5 | +/- | +/- | 3202 | 2965 | 47.3 |
|  | 100 | 82.0 | 67.5 | 64.5 | 65.0 | 61.5 | 64.0 | 64.0 | 64.0 | 33 | 56.5 | 44.5 | 46.5 | +/- | +/- | 2963 | 2842 | 42.8 |
|  | 250 | 78.5 | 56.0 | 51.5 | 52.0 | 39.5 | 48.5 | 48.5 | 48.5 | 23 | 42.0 | 38.5 | 35.5 | - | - | 2928 | 2797 | 37.9 |
